# Demonstrating LGBTQ+ Affirmative Practice in Groups:: Developing Competence through Simulation-Based Learning

**DOI:** 10.1007/s10615-022-00850-2

**Published:** 2022-08-16

**Authors:** Shelley L. Craig, Gio Iacono, Lauren McInroy, Alexa Kirkland, Rachael Pascoe, Toula Kourgiantakis

**Affiliations:** 1 Factor-Inwentash Faculty of Social Work, University of Toronto, 246 Bloor St. W, M5S 1V4 Toronto, ON Canada; 2 School of Social Work, University of Connecticut, Hartford, CT USA; 3 College of Social Work, The Ohio State University, Stillman Hall, 1947 College Rd, Columbus, OH USA

**Keywords:** LGBTQ+, Competence, Teaching, CBT, Simulation, Groups

## Abstract

Lesbian, gay, bisexual, transgender, queer, and other sexual and/or gender minority (LGBTQ+) populations experience significant mental and behavioral health disparities. Social workers are uniquely positioned to address these vulnerabilities. However, clinical graduate education has not effectively promoted or taught competent practice with LGBTQ+ populations. This qualitative study details the foundational competencies required for affirmative practice in group therapy with LGBTQ+ populations and describes a simulation-based learning activity designed to develop these competencies in graduate students. The following themes were identified as critical to affirmative practice, as identified through student reflections on their simulation-based learning experiences: deeply engaging in a strengths-based stance, keeping the group in group therapy, avoiding the expert trap, and managing identity assumptions. Implications for clinical social work education and practice are discussed.

**Introduction**.

Lesbian, gay, bisexual, transgender, queer, and other sexual and/or gender minority (LGBTQ+) populations experience mental and behavioral health disparities (Borgogna et al., 2019). When compared to their cisgender, heterosexual counterparts, LGBTQ+ individuals report disproportionately high rates of stress-associated concerns—such as depression, anxiety, and suicidality—due to the discrimination that results from their minoritized status in society (Meyer, 2003; Russell & Fish, 2016). Given the scope of their behavioral health needs, clinical social workers require basic competence in affirmative practice (a therapeutic approach that asserts the value of LGBTQ+ identities and experiences in a context of structural inequities) to work with LGBTQ+ populations (Alessi, 2013). Despite an increase in field LGBTQ+ specific training such as cultural competence (Kaiafas & Kennedy, 2021), positive space training (Haghiri-Vijeh et al., 2020), and for specific populations (e.g. gerontologists; Smith et al., 2019), many graduate clinical programs continue to have gaps in their explicit LGBTQ+ curricula, particularly related to applied skill development. Consequently, this paper details the qualitative results of a competency-based simulation-based learning (SBL) activity in a graduate social work course, which highlights the importance of experiential education in the development of affirmative clinical skills with LGBTQ+ populations, specifically in group therapy. This research sought to articulate: (1) clinical competencies for affirmative group therapy, (2) the utility of group based SBL in teaching affirmative practice, and (3) specific affirmative practice competencies identified by students participating in the SBL.

## Background

Preparing graduate students to provide counseling services to LGBTQ+ populations is critical, as LGBTQ+ individuals are present in all health systems and practice settings. It is estimated that 11 million LGBTQ+ adults and 3.5 million LGBTQ+ youth reside in the United States (Williams Institute, 2020), yet few services effectively and affirmatively serve these populations (Nelson et al., 2021). LGBTQ+people report that they have been subjected to discrimination and stigma from mental health service providers (Human Rights Watch, 2018). In a nationally representative study, 8% of LGB and 29% transgender clients report that they were refused care and an additional 9% of LGB and 21% of transgender reported being treated harshly by healthcare providers (Mirza & Rooney, 2018). Further, 18% of LGBQ (22% of transgender) adults avoided seeking healthcare due to anticipating discrimination (Casey et al., 2019). In behavioural health specifically, 24% of LGBTQ+ clients described their care as both negative and positive and 10% identified only negative experiences (Israel et al., 2008). Clients articulated counselling specific microaggressions from their psychotherapists including the assumption that all their problems stem from their sexual or gender identities, stereotypical assumptions, heteronormativity, and warnings about the dangers of identifying as LGBQ (Shelton & Delgado-Romero, 2011). A study of LGBTQ+ youth found that seeking professional support was rarely utilized as a coping strategy due to a range of factors such as lack of feeling understood to outright discrimination (Craig et al., 2017). Tailored training can positively impact attitudes and behaviors towards LGBTQ+ populations, which can contribute to reduced treatment disparities. However, increased knowledge is not sufficient. Clinicians may score high in knowledge acquisition, but still display biases and engage in harmful behaviors while providing care (Weeks, et al., 2018). Therefore, training efforts should integrate strategies to apply knowledge through the integration of experiential activities.

Graduate education aims to foster competent counselors who can effectively respond to the needs of vulnerable populations (Lee et al., 2020; Craig et al., 2017). Readiness to practice is typically understood as social work students’ final stage of competency development, yet many graduate students report not feeling ready to counsel LGBTQ+ individuals (Craig et al., 2016a). For example, a high percentage of LGBTQ+ identified social work students (n = 1018) have expressed not feeling adequately prepared for practice with LGBTQ+ communities, while indicating that their non-LGBTQ+ (i.e., cisgender, heterosexual) counterparts may be even less prepared (Craig et al., 2016). Greater readiness to practice with LGBTQ+ populations was associated with more support for LGBTQ+ students, skillful faculty discussion of LGBTQ+ issues, and comprehensive inclusion across the curriculum. Further, while foundational LGBTQ+ content is often included in courses, opportunities for applied clinical practice with LGBTQ+ populations is limited. Although in social work, the field placement is considered critical to develop competence, with the Council on Social Work Education referring to it as the ‘Signature Pedagogy’ (Wayne et al., 2010), the reliance on the field to cultivate clinical skills does not account for the issues that impede effective training. For example, a lack of time for supervision (Bogo et al., 2020), supervisor discomfort with LGBTQ+ issues or discriminatory organizational policies (Craig et al., 2016b) have an impact on field placements. Thus, emerging research has argued for more comprehensive graduate education that integrates opportunities to apply clinical skills in the classroom (Lee et al., 2020).

## Affirmative Practice

LGBTQ+ affirmative practice is an overarching framework for clinical practice that promotes positive self-regard and resilience and addresses the impact of discrimination on LGBTQ+ clients (Alessi, 2013; O’Shaughnessy & Spier, 2018). Affirmative practice also “appreciates the complexities inherent in the clinical care of this population” (Edwards-Leeper et al., 2016, pg. 166). For instance, clinicians should assist LGBTQ+ people to explore the contribution of structural factors such as transphobia on their negative feelings while focusing on their strengths and coping (Craig et al., 2021; Pachankis 2018). Overall, affirmative practice contributes to the improved mental health of LGBTQ+ clients through a strengthened therapeutic alliance (Alessi et al., 2019).

There has been some articulation of the critical components of affirmative care in the literature. The Association for Lesbian, Gay, Bisexual, and Transgender Issues in Counseling (ALGBTIC) state that competence includes addressing discrimination while recognizing how this stigma affects the development of LGBTQ+ people across the lifespan (ALGBTIC, 2012). As clients disclose experiences of discrimination, counselors should foster empowerment and support clients who may be grappling with their LGBTQ+ identities by engaging knowledge, skills, and attitudes that support affirmative language, and understanding that identities may shift over time (ALGBTIC, 2012). Clients perceive a stronger therapeutic relationship if their clinician had previous knowledge of their particular LGBTQ+ identity and conveyed authenticity in their application of affirmative practice (Kelly et al., 2015). Affirmative skills can be strengthened when clinicians practice reflexivity and examine their own privilege and biases (Oranksy et al., 2019). A competent clinician not only understands affirmative practice but can flexibly apply these practices to the needs of their specific client (O’Shaughnessy & Spier, 2018).

## Competencies for Social Work Practice with Groups

LGBTQ+ clients are often served in group counseling formats due to the effectiveness of groups for this population and the relative ease of implementation. Furthermore, given the importance of increasing social connectedness for LGBTQ+ people, group counseling is particularly suitable (Craig et al., 2020). The International Association for Social Work with Groups (IASWG, 2015) highlights core knowledge for competent practice with diverse groups (e.g., knowledge of groups behavior, individuals, and the function of a group therapist) (MacGowen et al., 2017). Group counselors should strive to utilize affirmative practice skills by engaging such strategies as creating a welcoming space, screening potential members, and requesting pronouns which establishes the therapeutic alliance and promotes participant validation and group cohesion (ALGBTIC, 2012).

Fostering mutual aid—a form of group therapy facilitation that shifts the emphasis of providing help and care from the social worker to the group members—is critical (Schwartz, 1961). To do so, clinicians should express empathy and openness toward group members’ diverse identities and stories while simultaneously fostering an environment in which conflict is addressed and members provide each other support (Knight & Gitterman, 2018). A group counsellor should be open to receive disclosures from participants about their identities and oppression while avoiding the pressure for all group members to participate in disclosing information equally (ALGBTIC, 2012). Given that students often feel unprepared to work with groups and lack opportunities to learn practical skills to competently facilitate group therapy (MacGowen & Wong, 2017), particularly with LGBTQ+populations (Craig et al., 2016a), creative approaches to clinical training are necessary.

## Simulation-Based Learning and Application with Diverse Populations

SBL is an innovative pedagogical approach to teaching clinical practice. Utilized in other healthcare disciplines (e.g., nursing, medicine; Pittiglio & Lidtke 2021), this experiential teaching method has been recognized for its effectiveness in promoting social work student competence (Bogo et al., 2021; Kourgiantakis et al., 2020). SBL provides students with the opportunity to engage in simulated client interactions with a trained actor (i.e., a standardized patient) in scenarios that resemble real-life practice situations (Craig et al., 2017). Drawing from the theoretical underpinnings of constructivism (Brown et al., 1989), adult learning (Knowles, 1984) and experiential learning theory (Kolb, 1984), research has noted that a key benefit of SBL compared to didactic clinical training is the opportunity to apply their understanding of competencies as they practice core clinical skills in a learning environment that allows students to take risks without posing any harm to real clients (Craig et al., 2020; Kourgiantakis et al., 2020). With a few limitations to implementing SBL (e.g., resource-heavy and second-best to live clinical interaction), it also permits instructors to teach and assess actual practice in the classroom without a sole reliance on self-report only (Beddoe et al., 2011). SBL provides the opportunity for students to go beyond exposure to a practice experience that increases clinical knowledge, skills, and self-awareness (Bogo et al., 2021).

Importantly, SBL permits the acquisition of skills to work with diverse clients (e.g., ethnically, sexually, and gender diverse; Craig et al., 2017; Logie et al., 2015). Schreiber & Minarik (2018) explored SBL in which undergraduate students participated in a group-based simulation with diverse clients (e.g., racially, politically) which helped students develop skills in managing difficult emotions, engaging in dialogue related to diversity, and challenging their assumptions and biases. Group-based SBL has also been used with social work graduate students to explore their own positionality as well as increase allyship and cultural humility (though a process of self-reflection and analysis of privilege) (Craig et al., 2021; Fisher-Borne et al., 2015).

The scant extant literature indicates SBL also provides opportunities for students to demonstrate core competencies with LGBTQ+ populations. A recent scoping review examining simulation in social work education found that only 4% of the studies reviewed (n = 52) directly incorporated culture and diversity issues and only one focused on LGBTQ+ clients (Kourgiantakis et al., 2020). Logie and colleagues (2015) utilized SBL to examine graduate students’ ability to work with a Black, lesbian youth disclosing their identities, and found that most students lacked confidence in their practice skills. Pittiglio & Lidtke (2021) found that a simulation scenario designed to deliver healthcare to a transgender adult resulted in improved attitudes, beliefs, comfort, and LGBTQ+ competence in undergraduate nursing students. Thus, this study explores the design and utility of group based SBL for graduate social work education focused on clinical competencies for affirmative practice and evaluated student learning through an analysis of their written reflections.

## Methods

A SBL activity was created and implemented with second year Master of Social Work (MSW) students (n = 25) in an elective course, *Social Work Practice with LGBTQ+ Populations*. Students ages ranged from 24 to 32 (*M* = 26); identified their gender identities as female (12), male (5), trans (5), gender diverse (3); their sexual orientations as straight (10), questioning (4), bisexual (3), gay (3), lesbian (2), pansexual (2), no response (1) and their racial and ethnic identities as white (9), Asian (5), Black (2), Indigenous (2), Latinx (1), no response (5). Study approval was granted by the University of Toronto Research Ethics Board which included student consent. The authors followed the emerging best practices for SBL (Kourgiantakis et al., 2020) and articulated the competencies for affirmative group therapy (Table 1) drawing on key sources (AALBTQ, 2012; IASWG, 2015)

**Table 1:** Affirmative Group Work Simulation Competencies

Affirmative Practice Skills
Demonstrate an affirmative stance (e.g., validation, support, and celebration of LGBTQ + identities, non-binary conceptualization of gender, verbal and non-verbal cues)
Use strengths-based language
Demonstrate recognition of the role of discrimination and minority stress
Respond affirmatively to clients’ experiences of discrimination
Elicit coping skills in clients experiencing discrimination
Group Skills
Establish a collaborative agenda and articulate group structure
Share responsibility for session structure and content with co-facilitator
Support the group process through facilitation (e.g., mutual aid)
Use summaries and feedback to respond to group members
Demonstrate group facilitation skills in pacing sessions and using time efficiently
Demonstrate interpersonal effectiveness (e.g., warmth, genuineness, empathy and understanding) within professional boundaries

Profiles of SGMY group members were developed to permit students to practice the identified competencies. These descriptions were drawn from the community implementation of AFFIRM, the first evidence-based, affirmative group intervention designed to address minority stressors encountered by LGBTQ+ youth and young adults (Craig et al., 2013; 2021). Designed in partnership with affirmative clinicians, graduate educators, and an LGBTQ+ youth advisory board, the profiles were designed to focus on basic affirmative practice competencies.

## Simulation description

This simulation scenario was set in a youth mental health center in a suburban area with two graduate students serving as group co-facilitators. The purpose of the group was to support the development of coping skills through an affirmative practice approach for LGBTQ + youth aged 16–19. The SBL was set to be the second session of an eight-session affirmative group intervention with six Standardized Patients (SPs), trained actors hired specifically to engage in clinical simulations, playing each of the specific youth roles (Table 2). No script was provided to represent real group dynamics more closely, but the SPs played their roles and the students worked to apply the basic affirmative competencies. The scenario was implemented during the final class of the course and modeled on the steps outlined by Bogo et al., (2014), utilizing SBL to assess competence, which included: (1) developing a brief written introduction (e.g., youth roles and expected competencies; (2) observing students conduct a session with SPs as clients; (3) providing feedback to students from the perspective of the SP (5 min, verbal), peers (fellow MSW students; 5 min, verbal), and a professional social worker (5 min, verbal); and (4) collecting a written reflection on their own performance from students. Prior to the final class, students received an explanation of the SBL process, the affirmative group competencies (Table 1) and background information on the client group participants played by SPs (Table 2). Students were matched randomly into co-facilitation dyads by the instructor using a random number generator in Microsoft Excel.

**Table 2:** Standardized Patient Roles: Affirmative Practice Clinical Group Simulation

Name	Identities and Relevant Information
*Bianca*	17-year-old in grade 11/ Uses she/her pronouns Identifies as pansexual and Genderqueer Lives with mother, father, younger sister, and younger brother Came out to her family three years ago and parents worry about what their communities may think (Filipino and Indian communities) Guidance counselor referred parents to Parents and Friends of Lesbians and Gays (PFLAG) and Bianca to group
*Tamara*	17-year-old in grade 12/ Uses she/her pronouns Identifies as Queer and came out one year ago to a teacher at school who referred her to the group Lives with her mother who identifies as Queer and is supportive, but a bit smothering. She does not want to tell her father she came out
*Annie-Claude*	18-year-old in grade 12/ Uses she/her pronouns Lives with her mother, father, and older sister (has another older sister who is away at university) Identifies as Queer and came out to her family when she was 12, but does not feel accepted by her family
*David*	19-year-old who is graduating from high school/ Uses he/him pronouns Lives with mother, father, twin brother and younger sister Identifies as gay and parents found out when a neighbor saw him with a boyfriend in grade 11 Parents do not think their community would be supportive (Jamaican) His boyfriend sees a SW who gave information about the group for David
*Alex*	19-year-old in first year at university Uses he/him pronouns Lives with father and older sister Identifies as trans and came out as male to his sister when he was 10 (after parents divorced) Counsellor from helpline recommended the group
*Sam*	18-year-old in grade 12/ Uses he/him pronouns Lives with his mother, younger brother and grandparents His father died when he was 5 in a car accident Identifies as bisexual, but has not come out because he worries about burdening his mother because she cares for his brother who has cerebral palsy He also thinks his grandparents would not understand He saw a flyer for a local mental health center and the counsellor referred him

The SBL group session was observed by the entire class. In addition to the instructor, an experienced affirmative clinical social worker was also present to provide feedback. Students were able to “pause” the scenario and seek feedback from the social worker or instructor. The session was structured into four phases: (1) review of introductions and group norms, setting a collaborative agenda, and reflection on the past week; (2) discussion on coping with discrimination; (3) skill building with a focus on one area (can include practice and rehearsal); and (4) group reflection, summarization of session and learning. MSW student dyads took on the role of co-facilitators for 15 min total; subsequent students built on the work of the previous facilitators with the first and last pairs of co-facilitators beginning and ending the group respectively. Debriefing occurred at multiple time points: (1) when students or facilitators would pause the activity; (2) directly following each student’s performance; and (3) at the very end of the scenario. The SPs also provided feedback from the client perspectives to students.

### Data Collection and Analysis

To investigate the impact of participation in the SBL activity on student learning, a qualitative study that elicited data from student assignments was designed (Kourgiantakis et al., 2020). Following the SBL, students completed an optional brief demographic survey and a written, graded reflective assignment of a maximum of six pages. Specifically, the assignment asked students to reflect on their SBL experience and discuss: (1) what they drew on from their social work education (2) how they engaged with diverse youth in a simulated group (3) what they learned about their strengths and challenges as a clinician (4) what is important for effective affirmative LGBTQ+ group practice and (5) how the SBL influenced their own learning goals related to LGBTQ+ practice. Student names were redacted by the instructor and all demographic data was collected independent of the assignment and aggregated prior to analysis. The key question guiding the analysis was: What are the skills or competencies for affirmative group practice identified by MSW students participating in a clinical SBL classroom activity? Three coders with a minimum of two years of affirmative group experience with LGBTQ+ youth used content analysis (Elo et al., 2014) to code the assignments. Coders met three times (2-hour meetings) to generate initial codes and themes. In keeping with similar counselling research (Moltu & Binder, 2014), lengthier direct quotations have been to provide rich examples of our findings and to additionally demonstrate the interpretative process.

## Results

Four key themes emerged from the student reflections that related to specific affirmative competencies and the utility of the group-based SBL: (1) deeply engaging in a strengths-based stance; (2) keeping the group in group therapy; (3) avoiding the expert trap; (4) and managing identity assumptions. These four themes are described below using direct student quotes.

### Theme 1: Deeply Engaging in a Strengths-Based Stance

Students articulated a deep engagement with the strengths-based stance foundational to affirmative practice. This was reflected through their grappling with addressing and affirming the challenges that SPs disclosed to the group, while simultaneously maintaining a strengths-based stance. Importantly, students recognized that a focus on strengths is not uncritical in its application, but rather integrates the broader context to intentionally validate the client experience. One student explained:

Learning to reframe and use a strengths-based approach has been a learning experience and I attempted to use these skills during the simulation as it is central to affirmative practice. I [found it could] be difficult to accurately reflect the challenges that the group members were facing, while still staying strengths-based. Despite potentially slipping into deficit-based language, I attempted to stay rather neutral… I tend to think that my critical mind is at odds with strengths-based [practice], [but] I believe it served me well. By being critical and aware of structural forms of oppression, I was able to reframe and reflect the discriminatory experiences that the group members faced in an affirming way. In my summary, I attempted to not only link their experiences, but to also remove the problem of not feeling accepted (or understood) from something inherent in themselves, to stemming from homophobic or transphobic experiences. I will work on my strengths-based language, but I will attempt to see how my critical mind can aid, rather than hinder, that process.

Some students found that they were able to successfully reframe negative comments and create opportunities for engagement through a strengths-based approach. One student stated:

I channeled enthusiasm and affirmative warmth that engaged more vocal and quieter group members … I was also able to think on my feet and reframe some group members’ dysfunction-related statements (“How is forgetting a good thing?”) toward a strengths-based perspective (“Forgetting can provide an opportunity to refresh old information and also learn something new”). Taking a strengths-based view enabled me to build rapport with my group members and—hopefully—help them see the potential for positivity and resilience in things that have traditionally carried negative or dysfunctional connotations (e.g., forgetting).

### Theme 2: Keeping the Group in Group Therapy

Students noted that the group modality could also facilitate strengths-based clinical approaches by providing opportunities for facilitators to affirm individuals while modeling that behaviour and engaging the group. By managing the dynamics of the virtual group, students recognized the importance of strengthening group cohesion through the cultivation of mutually affirming interactions.

Some examples of how I did move things forward was helping connect members especially in regards to their LGBTQ experiences. When [SP] Alex shared his experiences of transphobia at school, I responded, asking if the others had similar experiences and ideas for coping skills? The purpose of this was to strengthen group cohesion, build confidence of participants and expand healthy coping skills.

The above student reflected that there is pressure for the clinician to take responsibility for the therapeutic relationship and support among group members however, providing space for the LGBTQ+ group members to affirm each other’s experiences is powerful and fosters group cohesion. Another student reinforced this emphasis on group-based support:

This simulation also taught me more about the importance of mutual aid. One of the most powerful moments occurred when Bianca (a group member) offered to support David by connecting him with a social worker. This gave Bianca reason to return the following week, and incentive for David to return as well. Bianca likely felt good about helping, making this a positive and meaningful experience for her. David likely felt supported and validated. As a facilitator, I am quick to feel the responsibility of providing as much support as possible to participants; however, I should not forget nor discount the support that can exist between members.

Students agreed that engaging the group dynamics and paying attention to non-verbal communication was key when affirming the experiences of the group.

I engaged the group dynamics to try to move the group toward more group cohesion, which plays a major role in moving forward with goals in therapeutic group work. [I] attended to members’ nonverbal signals… to find opportunities to verbally engage quieter group members. I made a decision to call on [SP] Sam in a gentle way, as it seemed to be a relatively safe and welcoming moment for him to be given an explicit opportunity to verbally engage. In the end, he took the opportunity and verbally engaged with the group, which demonstrated to the group that even quiet members had something to contribute and demonstrated to Sam that it was a space where he might be able to take risks.

However, students also identified ways in which they did not focus on the group or clung to their own agenda, thereby missing out on opportunities to develop the group dynamic.

I exhibited tunnel vision in my efforts to stick to the didactic agenda (i.e., do a check-in question then review group norms), I totally missed some of my group members’ nonverbal cues—including signals of potential distress. It is important to balance practice and content by flexibly integrating both didactic material and emotional processing in a group session; this is a skill that I will work on improving.

Other students recognized that they focused too much on the individual, rather than creating opportunities to create member-to-member interactions between the group members.

My approach was akin to individual therapy rather than group therapy. A more effective approach would be volleying open-ended questions back to the group, asking, “what does the group think about these strategies?” Not only would these techniques align well with the group dynamic, but they would also make the group feel more like a cohesive collective – not a “room” with isolated silos.

### Theme 3: Avoiding the Expert Trap

Students noted that they used approaches that were incongruent with competent practice. For example, to mask their own emotional discomfort, they avoided the real issues the youth were bringing, instead using academic jargon to appear intelligent to their peers. This insistence on their own agenda and performance compromised the group process.

In retrospect, I see myself (over) using my knowledge to minimize my anxiety and mask the insecurity I was feeling in the group… I tried to appear like “the expert” and got in my way. Instead of exploring the members’ feelings around “feeling like a burden” or homophobia, I used academic jargon (cisnormativity, etc.). I was focused on impressing the group (and my colleagues watching) when I should have focused on affirming, validating, and listening to the youth. In an actual group session, I fear that my use of academic language [would make] some members feel isolated and (re)traumatized for not feeling “smart enough” to understand what I am trying to convey. Rather than using complex terminology to help members externalize their problems, I should have focused on the feelings homophobia elicits … [Then] I can use affirming statements/language to validate members’ experiences and use externalizing conversations to talk about skills or coping strategies that may help them when those feelings emerge.

Further, despite having time to plan together to prepare for the SBL, some students decided to try to control the dynamic by disregarding the cofacilitation opportunity.

It was like I didn’t have a cofacilitator as I did not engage with her but rather “took over”. When the simulation started, I initially felt anxious. I had a set plan for how I wanted the group session to go – and I wanted everything done perfectly, under my control. Rather than settling in my anxiety, I tried using the adrenaline I accumulated to appear energized. I felt insecure about my ability to facilitate the session. These feelings, coupled with the context of the simulation, were overwhelming, and instead of taking a moment to pause and share the reins, I tried to maintain control by ignoring my emotions and her.

### Theme 4: Managing Identity Assumptions

Despite many students verbally disclosing their own identities as members of the LGBTQ+ community, it is notable that some students learned to locate themselves and to not to assume shared experience by simply sharing one aspect of their identity with the group. Through self-examination, one student identified a challenge with explicitly affirming the SPs.

Participating in this simulation as a co-facilitator was a very valuable experience which highlighted the importance of… affirming LGBTQ identities to help with their healing … Upon reflection, I recognize an area for my own growth. During the simulation, when the participant, [SP] Alex, shared his experiences of transphobia, I did not explicitly affirm these experiences for him. In thinking about my experiences in the queer community, I assumed that Alex would know we all agreed that his experience was a product of transphobia and that we do not support this behavior. This assumption however, needed to be vocalized and further processed for Alex to build skills in coping with transphobia. This is an opportunity to externalize their mental health symptoms and identify how transphobia is impacting wellbeing. I will be much more explicit in affirming LGBTQ experiences.

Other students struggled to separate their own painful experiences from those of the group.

Discrimination has impacted me as a racialized member of the LGBTQ+ population. These events have also made me mindful of the complex ways discrimination can operate in someone’s life. When working with youth I feel like it offers me space where I feel as though I am the “parent”, where offering strategic life skills to garner protection of my clients is essential. My own experiences with discrimination and the need to protect youth are relevant to LGBTQ+ populations but add pressure to my clinical skills by focusing too heavily on what people will think and if my young clients are feeling physically and emotionally safe. Which takes attention away from the presenting issue and client priority.

Another student identified their need for self-disclosure to manage assumptions.

I attempted to use inclusive language and felt that some level of self-disclosure was appropriate to achieve that goal; by using a lot of “we” language in my empathic reflections, I was revealing to the group members that I was also LGBTQ + to minimize the client-facilitator divide.

A different student did not disclose and that also impacted their experience. This tension around identity disclosure contributed to some challenges during the SBL but led to reflective insights.

In the moment, I chose not to disclose yet I felt the tension of the representation of a false self in not disclosing to the group as I recognize the significance of invisible identities in creating braver spaces as well as the tension and stigma attached to disclosure.

Other students identified increased awareness about their own practice. This student discussed the way they analyze their personal experiences as someone who identifies as LGBTQ+ and how lacking a critical and reflexive lens can hinder group progress.

I came into this simulation thinking I would have the advantage in every activity because I am queer and non-binary. Although my sexual/gender identities inform my practice, I need to be mindful that my experiences are mine and may not reflect the realities of my clients. This simulation truly humbled me as I realized I have a lot more learning to do.

A heterosexual, cisgender student also identified learning related to assumptions.

Initially, I was very worried that I could not facilitate these groups as I am not LGBTQ + but I realized that I could help create a safer space where they could learn from one another.

Another student recognized that the experiences of the youth clients were different than their own. Checking their own assumptions assisted the student to practice affirmatively.

I am a member of the LGBTQIA community, this does not mean that I have automatic insight into what a client is going through. When I was a teenager, I was years away from accepting my sexuality, and even further from coming-out. Many of these young clients were already out, exploring their sexuality, developing relationships, and even brave enough to seek formal therapeutic support in the form of this group. If I were to assume that I already understood these youth simply because I’m gay too, I would be overlooking so much of the obvious strength and resilience they bring. I would also be at risk of oversimplifying their states of mental health and thinking they didn’t need clinical support because I would be comparing with my own experience. Furthermore, it’s possible I would project the negative experiences I had being in the closet by perhaps over-reassuring them and making them feel life was worse for them than it is. This experience reminded me to listen to each person’s story through as little personal filter as possible.

Overall, many students recognized the importance of affirmative statements and interventions, but they also noted that they needed more practice to develop these skills to ensure they were effectively and clearly delivered to clients.

## Discussion

Given the right of LGBTQ+ populations to access high quality care for their mental health, graduate education should support the development of competent affirmative clinicians. This study suggests that SBL offers a learning experience that highlights the importance of an affirmative competencies in practice with LGBTQ+ populations and reinforce the importance of opportunities to apply students’ classroom knowledge to the clinical environment using teaching methods that permit students to practice with no harm to real clients (Craig et al., 2017). Study findings identified themes surrounding the utility of SBL in teaching affirmative practice (i.e., deeply engaging in a strengths-based stance, keeping the group in group therapy) and specific learning that students obtained (i.e., avoiding the expert trap, and managing identity assumptions), which align with basic affirmative practice competencies.

Engaging strengths during affirmative groups was identified as an important competency that was strengthened during the SBL. The importance of acknowledging the potentially negative impact of minority stress on the lives of LGBTQ+ people while keeping the session focused on clients’ strengths is an important clinical skill that underlies affirmative practice (Alessi, 2013). Strengths-based interventions have been found to validate LGBTQ + identities and enhance self-esteem (Craig & Furman, 2018). Student reflections noted that utilizing the power of the group, engaging all the members and supporting them to affirm one another, was an important aspect of engaging in strengths-based and affirmative practice. LGBTQ+ youth particularly benefit from group therapy as it increases social support, which is critical to their wellbeing (Craig et al., 2021). The tendency to focus on one client at the expense of the group is often found with inexperienced clinicians but is an important group facilitation competence (IASWG, 2015).

Key competencies as identified by the students included practicing affirmatively, while also managing their own identities. Affirmative practice is not simply about being familiar with LGBTQ+ identities but rather deeply listening to and affirming LGBTQ+ clients within clinical interactions. Clinician self-disclosure, related to their identities and expertise, must be handled carefully to ensure the focus remains on the clients’ needs and safety. These types of self-disclosures can be helpful in the initial stages of joining with a client but can be a barrier to group progress if counsellors overidentify with clients and focus on their own needs. Sunderani & Moodley (2020) found that there is a greater tendency to self-disclose when there is a shared cultural similarity and suggest counselors consider disclosure with a lens of curiosity about the client instead of overidentification due to shared challenging experiences. Similarly, students’ false sense of awareness about LGBTQ + populations (the expert trap) has a negative impact on the quality of care that transgender patients receive from nurses (Maruca et al., 2018). Students in this SBL tried to manage their anxiety, by engaging their expertise, however they often created a power dynamic in which they were the experts which, in turn, undercut the group process. Similar to this study, research on SBL has found that students learn a great deal about recognizing and managing their emotions (e.g., emotional literacy) when reflecting on their simulation experiences (Schreiber & Minarik, 2018).

## Implications for Education and Practice

These results underscore the importance of providing experiential affirmative practice and group facilitation education throughout clinical coursework. Enhancing confidence in instructors and opportunities for SBL is important to improve practice (Kourgiantakis et al., 2020). In this SBL, when faced with an applied learning experience, many students resorted to their knowledge of the LGBTQ+ community, rather than engaging their clinical skills. Students were more comfortable stating what they know than demonstrating what they could do, which resulted in ignoring the distress manifesting in the group. Affirmative practice, which can also integrate trauma-informed approaches, necessitates that social work clinicians be aware of their own assumptions and experiences and reactions to clients’ identities and link their responses to their clients’ lives (Alessi et al., 2019; Levenson et al., 2021). Developing this self-awareness has been identified as one of the first steps to providing competent care (Maruca et al., 2018) and emerged as important learning in student reflections. Kourgiantakis et al. (2020) described the value of using SBL to teach students to locate the self and critically reflect on the role of power, privilege, and oppression in the helping relationship. Teaching affirmative practice could integrate cultural humility and an exploration of how their identities and macro issues impact the client improvement (Fisher-Borne et al., 2015). The results of this study suggest that some LGBTQ+ identified students, in particular, may have struggled to manage their assumptions about client needs and at times projected their own experiences onto the clients, potentially resulting in overconfidence, countertransference, and non-affirming practice. Klein & Nakhai (2016) highlight the importance of analyzing one’s own assumptions about LGBTQ+ populations in a safe educational space to more effectively focus on client needs. The results of this study suggest that utilizing SBL is a beneficial approach in clinical social work courses.

This study has several limitations. Given this SBL was embedded in an elective course focused on practice with LGBTQ+ populations, students may have had more knowledge of LGBTQ+ issues, based on personal, clinical, and research experiences, than other students. Given the data source was a graded reflective assignment and students wanted to be perceived positively by the instructor, there is risk of social desirability bias. Although the SBL performance was not graded, the simulation reflection counted towards 20% of the students’ final grade. Students were encouraged to provide critical appraisals of their performance in their reflection papers and were graded on the depth of their analysis rather than their stance. Despite this, however, students may have been reticent to fully engage in the graded assignment. The study design only required the capture of voluntary demographics. The convenience sample, as well as the single course limit the findings’ utility.

## Conclusions

This study suggests clinical training should ensure that the explicit curricula include a focus on affirmative practice with LGBTQ + populations utilizing group based experiential teaching methods. The findings also explicitly point to the importance of aligning teaching with clearly defined competencies to train students to effectively practice with LGBTQ+ populations.
